# ﻿Three coralloid species of the genus *Trechispora* (Trechisporales, Basidiomycota) in China: two newly discovered taxa and one reported for the first time

**DOI:** 10.3897/mycokeys.99.109375

**Published:** 2023-09-07

**Authors:** Peng-Tao Deng, Jun Yan, Xiang-Fen Liu, Zheng-Mi He, Yuan Lin, Ming-Xin Lu, Ping Zhang

**Affiliations:** 1 College of Life Science, Hunan Normal University, Changsha 410081, China Hunan Normal University Changsha China; 2 Bureau of Forestry, Tongdao Dong Autonomous County, Huaihua, Hunan 418500, China Bureau of Forestry,Tongdao Dong Autonomous Huaihua China

**Keywords:** Coral fungi, Phylogenetic analysis, *Scytinopogon*, Taxonomy

## Abstract

Two new species of *Trechispora* indigenous to southern China, *T.laxa* and *T.tongdaoensis*, are described and illustrated, and the first record of *T.khokpasiensis* in China is reported. Molecular phylogenetic analyses of the concatenated nuclear rDNA ITS1–5.8S–ITS2 and nuclear large subunit sequences supported the inclusion of the three species within the *Trechispora* clade, together with species formerly classified in *Scytinopogon*. The new species are similar in micromorphology to species of *Trechispora* (as traditionally circumscribed) but are distinguished by having coralloid basidiomata. A key to the known coralloid *Trechispora* species in China is provided.

## ﻿Introduction

The genus *Trechispora* P. Karst was established by Karsten (1890) with *Trechisporaonusta* P. Karst as the type species. *Trechispora* is the largest genus in the order Trechisporales (Larsson 2007), which is highly diverse in morphology: stipitate, clavarioid, or resupinate basidiomata (Meiras-Ottoni et al. 2021; Sommai et al. 2023); smooth, grandinioid, odontoid, hydnoid, or poroid hymenophores; short cylindric basidia, clamped, with 2 or 4 sterigmata on the basidia; and smooth or variously ornamented basidiospores. In addition, ampullate septa is an important character in *Trechispora* (Bernicchia and Gorjón 2010, Ordynets et al. 2015, Meiras-Ottoni et al. 2021, Liu et al. 2022). Calcium oxalate crystals usually accumulate on the mycelium or subhymenial hyphae, and the crystal morphology can be useful for species identification (Larsson 1994). Currently, approximately 90 species are accepted in *Trechispora* (Liu et al. 2022; Sommai et al. 2023). Consistent with previous studies (Birkebak et al. 2013), most *Trechispora* species are distributed in the tropics or subtropics (Chikowski et al. 2020). The placement of *Trechispora* in the order Trechisporales is supported by phylogenetic analyses of molecular data (Hibbett et al. 2007; Larsson 2007).

*Scytinopogon* Singer, erected by Singer (1945), has been assigned to several different families in the past: Clavariaceae Chevall (Corner 1970), Thelephoraceae Chevall (Donk 1964), and Gomphaceae (Maas Geesteranus 1962). The genus has also been suggested to be related to the Hydnodontaceae (Jülich 1981). Morphologically, *Scytinopogon* is characterized by clavarioid basidiomata with flattened and dense branches, which distinguish the species from *Trechispora*. However, phylogenetic analyses of molecular data indicate that *Scytinopogon* is nested within *Trechispora* (Hydnodontaceae), and no clear delimitation exists between the two genera. Because the name *Trechispora* has nomenclatural priority, *Scytinopogon* has been synonymized with *Trechispora*, thus rendering *Trechispora* a large, monophyletic genus (Meiras-Ottoni et al. 2021). To determine the correct name for *Scytinopogon* species, sequences and specimens for the type species were needed (Meiras-Ottoni et al. 2021). For this reason, to avoid misinterpretations in species delimitation within *Trechispora*, not all currently accepted *Scytinopogon* species have been transferred to *Trechispora*. *Scytinopogoncryptomerioides* W.R. Lin and P.H. Wang has recently been described from Taiwan (Lin et al. 2022).

During research on clavarioid fungi indigenous to southern China, two undescribed and one recently described coralloid *Trechispora* species were collected. Descriptions and illustrations of these three species are provided, and phylogenetic reconstructions based on nuclear rDNA ITS1–5.8S–ITS2 (ITS) and nuclear large subunit (LSU) sequences support the distinction of the new species and their placement in *Trechispora*.

## ﻿Materials and methods

### ﻿Specimen sources

Field work was conducted and specimens gathered by the authors from 2011 to 2022 in Hainan, Hunan, and Guangdong provinces, China. The habitat and morphological characters of fresh specimens were recorded in the field, including their dimensions and color. The fresh fruiting bodies were dried using heat or silica gel. The dried specimens were deposited in the Mycological Herbarium of Hunan Normal University (**MHHNU**), Changsha, China.

### ﻿Morphological observation

Macroscopic characteristics were mainly derived from record sheets and photographs. The colors cited in the descriptions are based on those of Kornerup and Wanscher (1978) and Ridgway (1912). Dried fruiting body sections were placed in 3% KOH solution containing 1% Congo red solution. Microscopic characters were observed from a small portion of dried hymenial tissue using a light microscope to observe the basidiospores (100×), basidia, and hyphae. Scanning electron microscopy (SEM) was conducted with a TESCAN CLARA Xplore 30 operating at 2 keV. Forty basidiospores of each specimen were randomly selected for measurement. The spore size is expressed in the form (a–) b–c (– d), where a and d are the minimum and maximum dimensions of spores, respectively, and b and c encompass the majority of the spore dimensions. The abbreviation [n/m/p] refers to n spores measured from m basidiomata of p specimens. In addition, the Q value represents the length: width ratio of basidiospores, and the Q_m_ value is the average Q ± standard deviation.

### ﻿DNA extraction, PCR amplification, and sequencing

Genomic DNA was extracted from dried specimens using the EZup Column Fungal Genomic DNA Extraction Kit (Sangon Biotech, Shanghai, China). A 20 mg sample of a dried specimen was ground to powder in liquid nitrogen in accordance with the manufacturer’s instructions. The primer pairs ITS4/ITS5 and LR5/LR0R were used to amplify the ITS and LSU regions, respectively (Vilgalys and Hester 1990; White et al. 1990; Gardes and Bruns 1993). The PCR amplification reactions were performed on an Eppendorf Mastercycler thermal cycler in a 25 µL volume containing 1 µL DNA, 2 µL primers, 9.5 µL ddH2O, and 12.5 µL 2× Es Taq Master Mix. The amplification procedure consisted of pre-denaturation at 94 °C for 4 min, then 32 cycles comprising denaturation at 94 °C for 40 s, annealing at 55 °C for 40 s, and extension at 72 °C for 1 min, followed by a final extension at 72 °C for 8 min, and held at 4 °C (Liu et al. 2022). The PCR products were separated by electrophoresis on a 1% agarose gel. An ABI 3730 DNA Analyzer (PerkinElmer Inc., USA) was used to sequence the PCR products. The newly generated sequences (seven ITS and seven LSU) were deposited in GenBank (Table 1).

### ﻿Alignment and phylogenetic analysis

The newly generated sequences were aligned with publicly available ITS and LSU sequences of *Scytinopogon* and *Trechispora* species from GenBank (see Table 1 for the accession numbers and sources of previously published sequences). Sequences for the corresponding regions from single accessions of *Brevicelliciumolivascens* K.H. Larss. and Hjortstam and *Brevicelliciumatlanticum* Melo, Tellería, M. Dueñas and M.P. Martín were used as the outgroup and included in the ITS+LSU sequence matrix. The ITS and LSU sequences were aligned using MAFFT v7.471 with default settings of gap openings and extension penalties (Katoh and Standley 2016). The final ITS+LSU dataset comprising 151 sequences and 1663 aligned positions (77 ITS and 74 LSU). It was assembled with SEQUENCEMATRIX v1.7.8 (Vaidya et al. 2011) and used for a multimarker phylogenetic analysis. A maximum likelihood (ML) analysis was conducted with RAxML v7.2.6 (Stamatakis et al. 2005, Stamatakis 2006) using the GTR+Gamma evolutionary model (Stamatakis et al. 2008). ML bootstrapping (BS) was performed with 1000 replicates. Bayesian inference (BI) was performed using MrBayes v3.2.7 (Ronquist and Huelsenbeck 2003); analyses were run for 2,000,000 generations using four Metropolis-coupled Monte Carlo Markov chains to calculate posterior probabilities (PP). FigTree 1.4.2 (Rambaut 2012) was used to visualize the tree files, which were edited using Adobe Photoshop CS6 (Adobe Systems Inc., USA).

**Table 1.** Details of the ITS and 28S rDNA sequences used for phylogenetic analyses. The sequences newly generated in this study are highlighted in bold, and all types marked with an asterisk.

Taxon	Voucher	GenBank No. (ITS)	GenBank No. (28S)	Geographical origin	References
*Trechisporaaraneosa*	KHL8570	AF347084	AF347084	Sweden	Larsson et al. (2004)
*T.bambusicola*	CLZhao3302	MW544021	MW520171	China	Zhao et al. (2021)
*T.bambusicola*	He3381	OM523405	OM339227	China	Liu et al. (2022)
*T.chaibuxiensis*	He5072	OM523408	OM339230	China	Liu et al. (2022)
*T.chaibuxiensis*	LWZ2017081434	OM523409	OM339231	China	Liu et al. (2022)
T.copiosa*	AMO422	MN701013	MN687971	Brazil	Meiras-Ottoni et al. (2021)
*T.copiosa*	AMO427	MN701015	MN687973	Brazil	Meiras-Ottoni et al. (2021)
*T.copiosa*	AMO453	MN701018	MN687975	Brazil	Meiras-Ottoni et al. (2021)
*T.confinis*	KHL11064	AF347081	AF347081	Sweden	Larsson et al. (2004)
*T.confinis*	LWZ2021092023b	OM523414	OM339235	China	Liu et al. (2022)
*T.constricta*	He5899	OM523417	OM339236	China	Liu et al. (2022)
*T.constricta*	LWZ2021092430a	OM523418	OM339237	China	Liu et al. (2022)
T.caulocystidiata*	FLOR56314	MK458772	–	Brazil	Furtado et al. (2021)
*T.crystallina*	LWZ201707292	OM523419	OM339238	China	Liu et al. (2022)
*T.crystallina*	LWZ 201710137	OM523420	OM339239	Vietnam	Liu et al. (2022)
*T.dimitiella*	Dai21181	OK298493	OK298949	China	Liu et al. (2022)
T.dimitiella*	Dai 21931	OK298492	OK298948	China	Liu et al. (2022)
*T.dealbata*	FLOR56183	MK458777	–	Brazil	Furtado et al. (2021)
*T.fimbriata*	He 4873	OM523424	OM339243	China	Liu et al. (2022)
*T.fimbriata*	He 6134	OM523425	OM339244	China	Liu et al. (2022)
*T.fissurata*	He6190	OM523427	OM339245	China	Liu et al. (2022)
*T.fissurata*	He6322	OM523428	OM339246	China	Liu et al. (2022)
T.foetida*	FLOR56315	MK458769	–	Brazil	Furtado et al. (2021)
*T.farinacea*	KHL 8451	AF347082	AF347082	Sweden	Larsson et al. (2004)
*T.farinacea*	KHL 8454	AF347083	AF347083	–	Larsson (2001)
*T.gelatinosa*	AMO824	MN701020	MN687977	Brazil	Meiras-Ottoni et al. (2021)
T.gelatinosa*	AMO1139	MN701021	MN687978	Brazil	Meiras-Ottoni et al. (2021)
T.havencampii*	SFSUDED8300	NR154418	NG059993	Africa	Desjardin and Perry (2015)
*T.hymenocystis*	KHL16444	MT816397	MT816397	Norway	Meiras-Ottoni et al. (2021)
*T.hymenocystis*	KHL8795	AF347090	AF347090	Sweden	Larsson et al. (2004)
T.khokpasiensis*	MMCR00009	MZ687107	MZ683197	Thailand	Sommai S et al. (2023)
*T.latehypha*	He5848	OM523446	OM339262	Sri Lanka	Liu et al. (2022)
*T.latehypha*	LWZ2017061116	OM523447	OM339263	China	Liu et al. (2022)
*T.longiramosa*	HG140168	OM523448	OM339264	China	Liu et al. (2022)
*T.longiramosa*	CH 19233	OM523449	–	China	Liu et al. (2022)
***T.laxa***	**MHHNU10379**	**OP959649**	**OP954660**	**China**	**This study**
T.laxa*	**MHHNU10714**	**OP959650**	**OP954661**	**China**	**This study**
*T.malayana*	Dai17876	OM523452	OM339265	Singapore	Liu et al. (2022)
*T.malayana*	He4156	OM523453	OM339266	Thailand	Liu et al. (2022)
*T.minispora*	AM170	MK328885	MK328894	Mexico	Yuan et al. (2020)
*T.minispora*	AM176	MK328886	MK328895	Mexico	Yuan et al. (2020)
*T.mollusca*	Dai 6191	OM523455	OM339269	China	Liu et al. (2022)
*T.mollusca*	Dai 11085	OM523457	OM339270	China	Liu et al. (2022)
*T.nivea*	LWZ201808043	OM523461	OM339273	China	Liu et al. (2022)
*T.nivea*	MAFungi74044	JX392832	JX392833	–	Telleria et al. (2013)
*T.papillosa*	AMO713	MN701022	MN687979	Brazil	Meiras-Ottoni et al. (2021)
*T.papillosa*	AMO714	–	MN687980	Brazil	Meiras-Ottoni et al. (2021)
T.papillosa*	AMO795	MN701023	MN687981	Brazil	Meiras-Ottoni et al. (2021)
*T.pallescens*	FLOR56184	MK458767	–	Brazil	A.N.M. Furtado et al. (2021)
*T.pallescens*	FLOR56188	MK458774	–	Brazil	A.N.M. Furtado et al. (2021)
*T.aff.pallescens*	RL 115	MK328887	MK328896	Mexico	Unpublished
*T.aff.pallescens*	RL 132	MK328889	MK328898	Mexico	Unpublished
*T.aff.pallescens*	RL 133	MK328890	MK328899	Mexico	Unpublished
*T.robusta*	FLOR 56179	MK458770	–	Brazil	A.N.M. Furtado et al. (2021)
*T.sanpapaoensis*	MMCR00124.1	MZ687109	MZ683200	Thailand	Sommai S et al. (2023)
T.saluangensis*	MMCR00260	MZ687104	MZ683201	Thailand	Sommai S et al. (2023)
*T.saluangensis*	MMCR00261	MZ687105	MZ683202	Thailand	Sommai S et al. (2023)
*T.scabra*	FLOR56189	MK458773	–	Brazil	A.N.M. Furtado et al. (2021)
*T.sinensis*	He3714	OM523464	OM339274	China	Liu et al. (2022)
*T.sinensis*	He4314	OM523465	OM339275	China	Liu et al. (2022)
*T.stevensonii*	MAFungi70645	JX392843	JX392844	–	Telleria et al. (2013)
*T.stevensonii*	MAFungi70669	JX392841	JX392842	–	Telleria et al. (2013)
*T.subfissurata*	LWZ2019061348	OM523491	–	China	Liu et al. (2022)
*T.subfissurata*	He3907	OM523490	OM339298	China	Liu et al. (2022)
***T.khokpasiensis***	**MHHNU07529**	**ON897819**	**ON898005**	**China**	**This study**
***T.khokpasiensis***	**MHHNU10662**	**ON897822**	**ON898008**	**China**	**This study**
***T.khokpasiensis***	**MHHNU10670**	**ON897823**	**ON898009**	**China**	**This study**
T.thailandica*	He4101	OM523499	OM339307	Thailand	Liu et al. (2022)
*T.thailandica*	He 4112	OM523500	OM339308	Thailand	Liu et al. (2022)
*T.thelephora*	URM85757	–	MH280001	Brazil	Chikowski et al. (2020)
*T.thelephora*	URM85758	–	MH280002	Brazil	Chikowski et al. (2020)
T.torrendii*	URM85886	MK515148	MH280004	Brazil	Chikowski et al. (2020)
*T.torrendii*	URM85887	–	MH280005	Brazil	Chikowski et al. (2020)
T.tongdaoensis*	**MHHNU11083**	**OP959651**	**OP954662**	**China**	**This study**
***T.tongdaoensis***	**MHHNU11086**	**OP959652**	**OP954663**	**China**	**This study**
T.termitophila*	AMO396	MN701025	MN687983	Brazil	Meiras-Ottoni et al. (2021)
*T.termitophila*	AMO893	MN701026	MN687984	Brazil	Meiras-Ottoni et al. (2021)
*T.termitophila*	AMO1169	MN701028	MN687986	Brazil	Meiras-Ottoni et al. (2021)
*T.tropica*	LWZ2017061314	OM523502	OM339310	China	Liu et al. (2022)
*T.tropica*	LWZ2017061316	OM523503	OM339311	China	Liu et al. (2022)
Scytinopogoncryptomerioides*	0906RK10-23	–	OK422242	China	Lin et al. (2022)
*Brevicelliciumatlanticum*	LISU178566 9065IM	HE963773	HE963774	Portugal	Telleria et al. (2013)
*B.olivascens*	MAFungi23496	HE963787	HE963788	Spain	Telleria et al. (2013)

## ﻿Results

### ﻿Phylogenetic analyses

**Figure 1.**
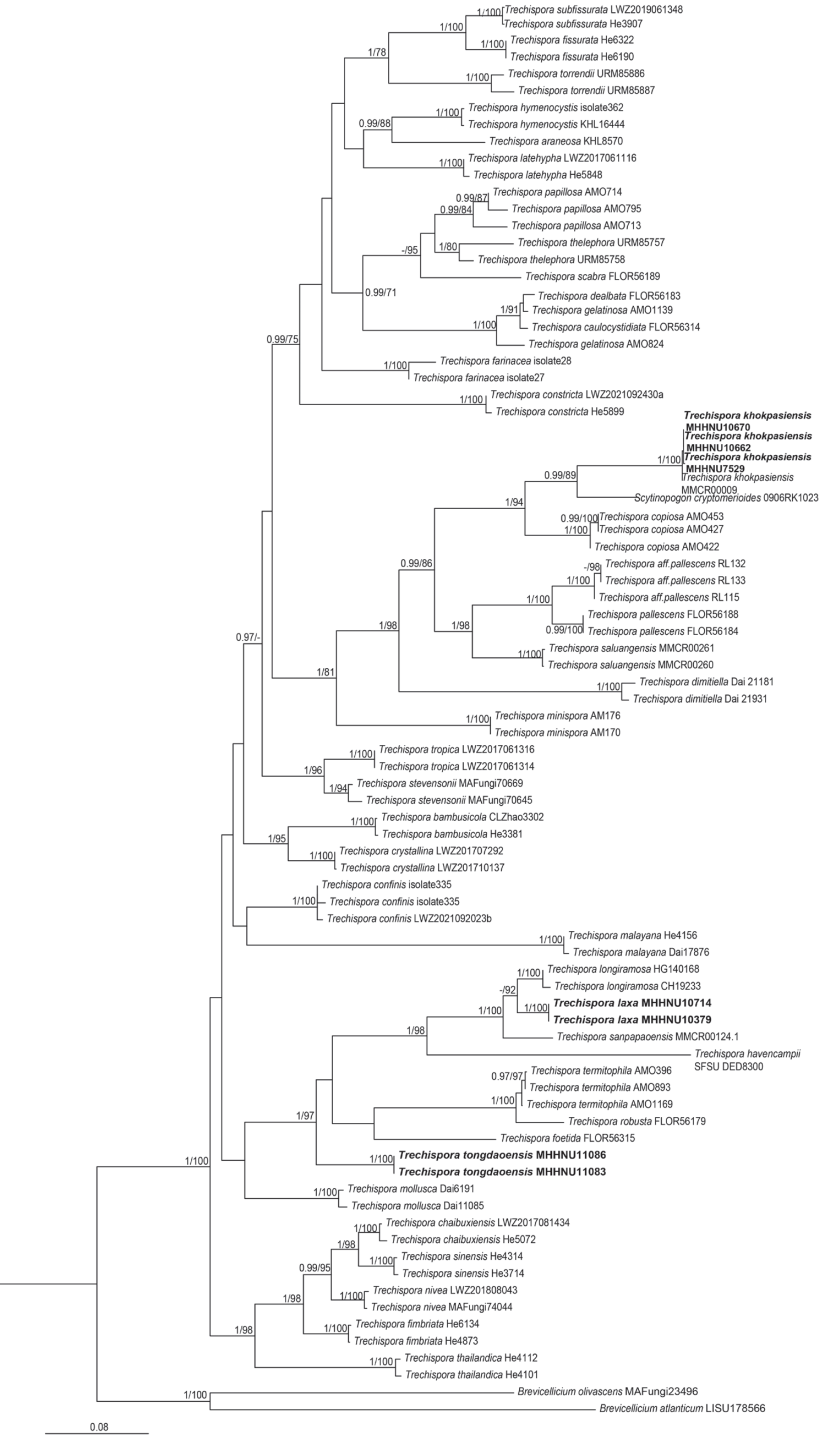
Phylogenetic relationships of *Trechispora* species inferred from a concatenated ITS and LSU sequence dataset under the maximum likelihood optimality criterion. Bayesian posterior probabilities (PP) > 0.95 and bootstrap values (BS) >70% are reported at the nodes (PP/BS); “–” indicates that the support value was less than the respective threshold. The two newly described species and one newly recorded species from China are highlighted in bold.

The phylogeny derived from the ML analysis of the concatenated ITS+LSU dataset, with both PP and BS support values, is shown in Fig. 1. The BI phylogeny (not shown) was very similar in topology and branch support to the ML tree. The ML and BI analyses resolved that the three species collected from southern China each formed a monophyletic lineage within the genus *Trechispora* with high statistical support values. The species of *Trechispora* and *Scytinopogon* were intermixed, which was consistent with previous phylogenetic studies (Meiras-Ottoni et al. 2021; Chikowski et al. 2020). *Trechisporalaxa* and *T.tongdaoensis* were closely related to the species *Trechisporahavencampii* (D.E. Desjardin and B.A. Perry) Meiras-Ottoni and Gibertoni, *Trechisporarobusta* (Rick) S.L. Liu and L.W. Zhou, *Trechisporafoetida* (A.N.M. Furtado and M.A. Neves) S.L. Liu and L.W. Zhou, *Trechisporalongiramosa* S.L. Liu, G. He, L. Chen Shuang and L.W. Zhou, *Trechisporasanpapaoensis* Pinruan, Sommai and Khamsuntorn, and *Trechisporatermitophila* Meiras-Ottoni and Gibertoni. *Trechisporakhokpasiensis* Pinruan, Sommai and Khamsuntorn, *Trechisporacopiosa* Meiras-Ottoni and Gibertoni, and *Scytinopogoncryptomerioides* were grouped in a well-supported subclade (PP 0.99, BS 86%). These results strongly supported the phylogenetic distinction of *T.laxa* and *T.tongdaoensis* from other species within the *Trechispora* clade.

### ﻿Taxonomy

#### ﻿ Trechispora khokpasiensis

Taxon classificationFungiTrechisporalesHydnodontaceae

Pinruan, Sommai & Khamsuntorn

78E95334-3CBC-52E4-8ABC-2E7C6124D647

Figs 2 , 3 , 4

##### Basidiomata.

Clavarioid, scattered or fascicled, 25–30 mm tall, 15–36 mm broad, chalk white (1A1), slightly yellow (2A2) with age, apices white (1A1), yellowish white (3A4) when dry. Stipe single, short and flattened, 10–15 × 3–4 mm, white. Branches flattened or palmate, palmately branched from flattened stipe, dense, 4–7 mm wide, polychotomous below, dichotomous towards apices, internodes becoming gradually longer, branches 6–8 mm diam, divided 3–5 times, apices cristate or flattened, blunt, axils V-shaped. Flesh white to pale yellow, waxy. Taste and odor unrecorded.

**Figure 2.**
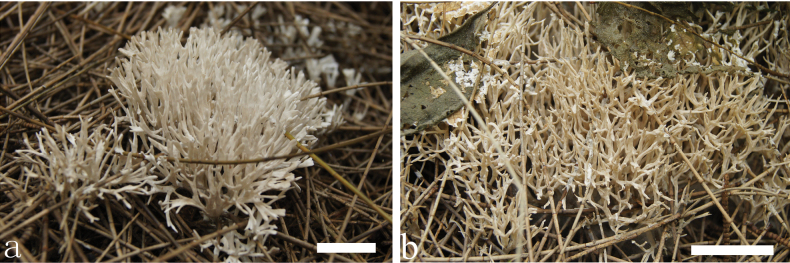
Basidiomata of *Trechisporakhokpasiensis* (MHHNU7529). Scale bars: 1cm.

##### Micromorphology.

Generative hyphae septate, clamped, interwoven, smooth, thin-walled, hyaline; tramal hyphae parallel arranged, 2–4 μm wide, smooth, thin-walled, hyaline. Subhymenial hyphae branched and wide, 3–8 μm; ampulliform septa present in the hyphae, 5−6 µm wide. Basidia: approximately 22–28 × 5.5–8 µm with four sterigmata 3–4.5 µm long, hyaline, subcylindrical to clavate, slight constriction, clamp connection in base. Cystidia absent. Basidiospores [40/4/3] 5–6 (–6.5) × 3–4 μm [Q = 1.33–1.72(1.83), Q_m_ = 1.57 ± 0.16], ellipsoid, angular, finely verruculose or echinulate, hyaline, thin-walled, spines 0.5–1 μm long, apex slightly blunt; hilar appendage extremely small, obscured by spore ornamentation, inamyloid, contents usually uniguttulate.

**Figure 3.**
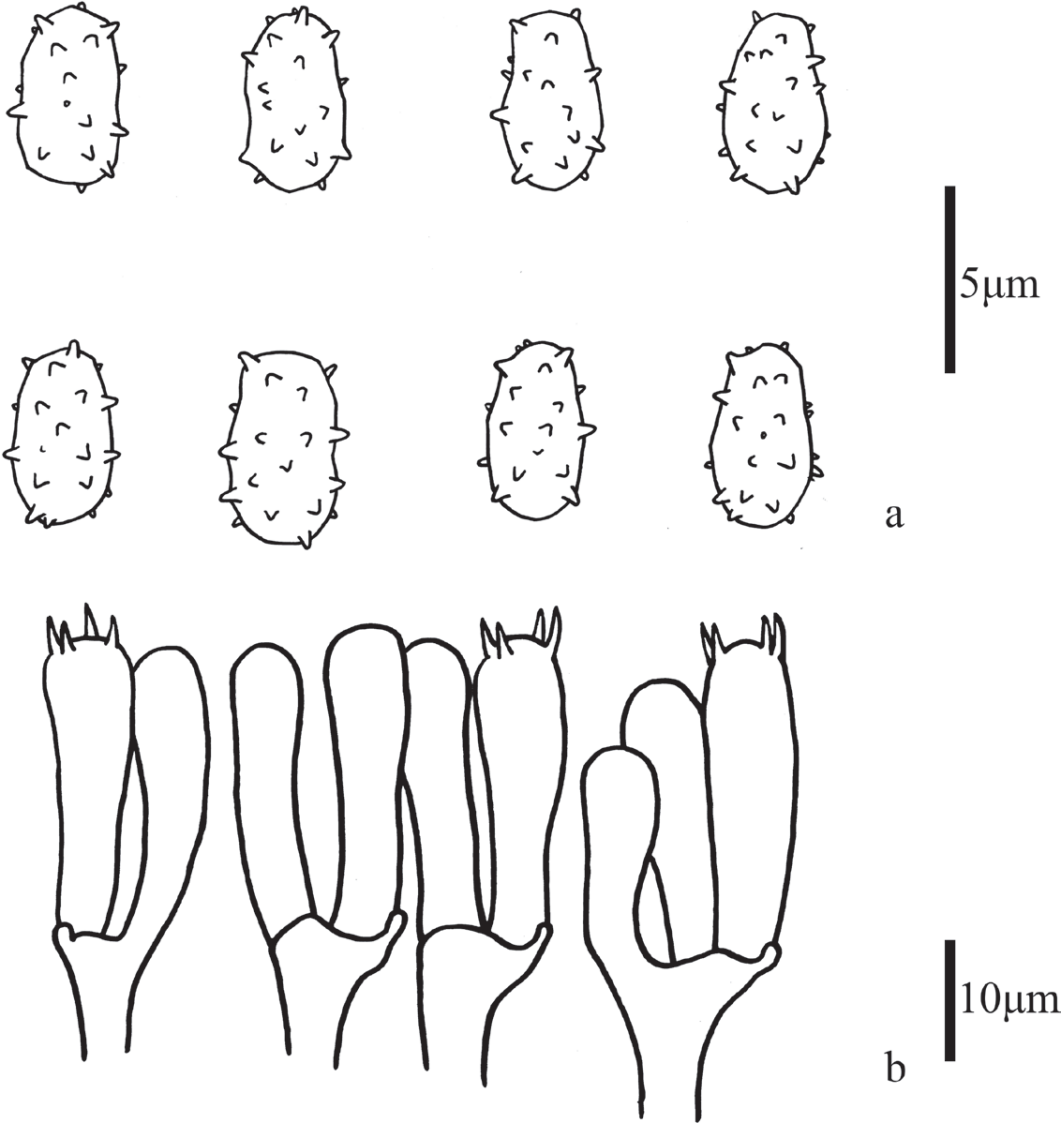
Microscopic features of *Trechisporakhokpasiensis* (MHHNU7529) **a** basidiospores **b** basidia.

##### Habit and distribution.

Solitary to caespitose, grows on humus in broadleaf forest or grows on soil; basidiomata generally occur in summer. Known from Thailand (Sommai et al. 2023), Laos and China.

**Figure 4.**
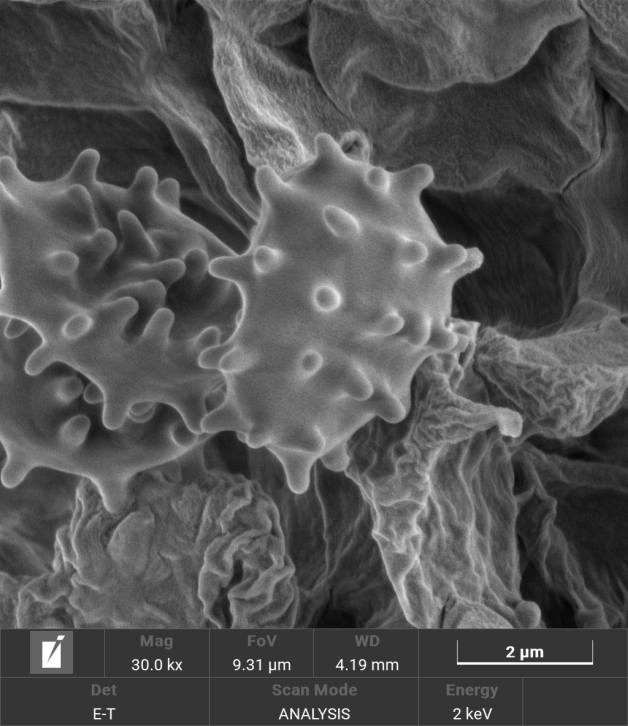
Scanning electron micrograph of basidiospores of *Trechisporakhokpasiensis* (MHHNU7529).

##### Notes.

*Trechisporakhokpasiensis* is mainly characterized by chalk-white basidiomata and flattened branches. *Trechisporapallescens* (Bres.) Singer is easily mistaken for *T.khokpasiensis* in the field on account of its similar size, shape, and color. However, the two species occur in different habitats: *T.khokpasiensis* grows in the humus layer on soil without any plant root association. *Trechisporachartacea* (Pat.) Gibertoni also has flattened, narrowly spathulate branches, grayish white in age, axils U-shaped, arising from scarce white mycelia on the soil. However, *T.khokpasiensis* differs in that the axils are V-shaped, the basidiomata are pale yellow with age, and it grows on dead branches and leaves. *Trechisporacaulocystidiata* is distinguished from *T.khokpasiensis* by having subglobose basidiospores and possessing caulocystidia.

In the present phylogenetic analyses, *Scytinopogoncryptomerioides* was close to *T.khokpasiensis*, but the two species differ in that *T.khokpasiensis* has relatively smaller basidia (22–28 × 6–8 μm vs. 35–42 × 5.5–6 μm in *S.cryptomerioides*). *Trechisporacopiosa* has similar branches to *T.khokpasiensis*, but in *T.copiosa* the branches are moderately open and the basidia are primarily 2–4-spored.

#### ﻿ Trechispora laxa

Taxon classificationFungiTrechisporalesHydnodontaceae

P. Zhang & P.T. Deng sp. nov.

79637F69-608E-585D-AC85-834FC1A4715F

MycoBank No: 846842

Figs 5 , 6 , 7

##### Diagnosis.

Differs from *Trechisporahavencampii* by the loose branches and 4-spored basidia.

##### Type.

China, Hainan Province, Baoting County, Qixianling Hot Springs National Forest Park, 18°70′24″N, 109°69′35″E, 300 m asl, 31 July 2021, leg. P. Zhang (holotype MHHNU10714).

##### Etymology.

*laxus* (Latin), loose, referring to the loose branching.

##### Basidiomata.

Solitary or scattered, fleshy consistency, 45–55 mm tall, 30–35 mm broad, fresh color (8B6–7), apices white when young but turning grayish purple (14B6) with age, drying pale grayish beige (4C3). Stipe single, white (1A1), 10–15 mm tall. Branches polychotomous from the stipe, dichotomous towards apices, not flattened, loose, divided 3–5 times, terminal branches relatively short and with color transitions to lilac, apices pale purple or white, acute, axils U-shaped. Taste and odor not recorded.

**Figure 5.**
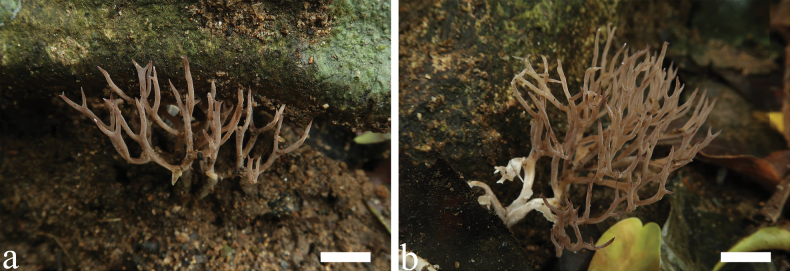
Basidiomata of *Trechisporalaxa* (MHHNU10714). Scale bars: 1 cm.

##### Micromorphology.

Context with parallel arranged hyphae, 2–4 μm wide; generative hyphae clamped, smooth, thin-walled, hyaline, no calcium oxalate crystals. Subhymenial hyphae branched and wide, 3–8 μm; ampullate septa present at the base of the stipe, up to 6–8 μm wide. Basidia 20–26 × 7–9 µm with four sterigmata 4–5 µm long and a basal clamp connection, hyaline, subclavate, barrel-shaped. Cystidia absent. Basidiospores [40/3/2] 5–6 × 3–4 μm [Q = 1.25–1.71(1.83), Q_m_ = 1.46 ± 0.16], ellipsoid, slightly irregular, inner side slightly concave, aculeate or finely verrucose, spines 1–1.5 μm long, apex not sharp but blunt; hilar appendage obscured by spore ornamentation; usually uniguttulate; hyaline, thin-walled, inamyloid.

**Figure 6.**
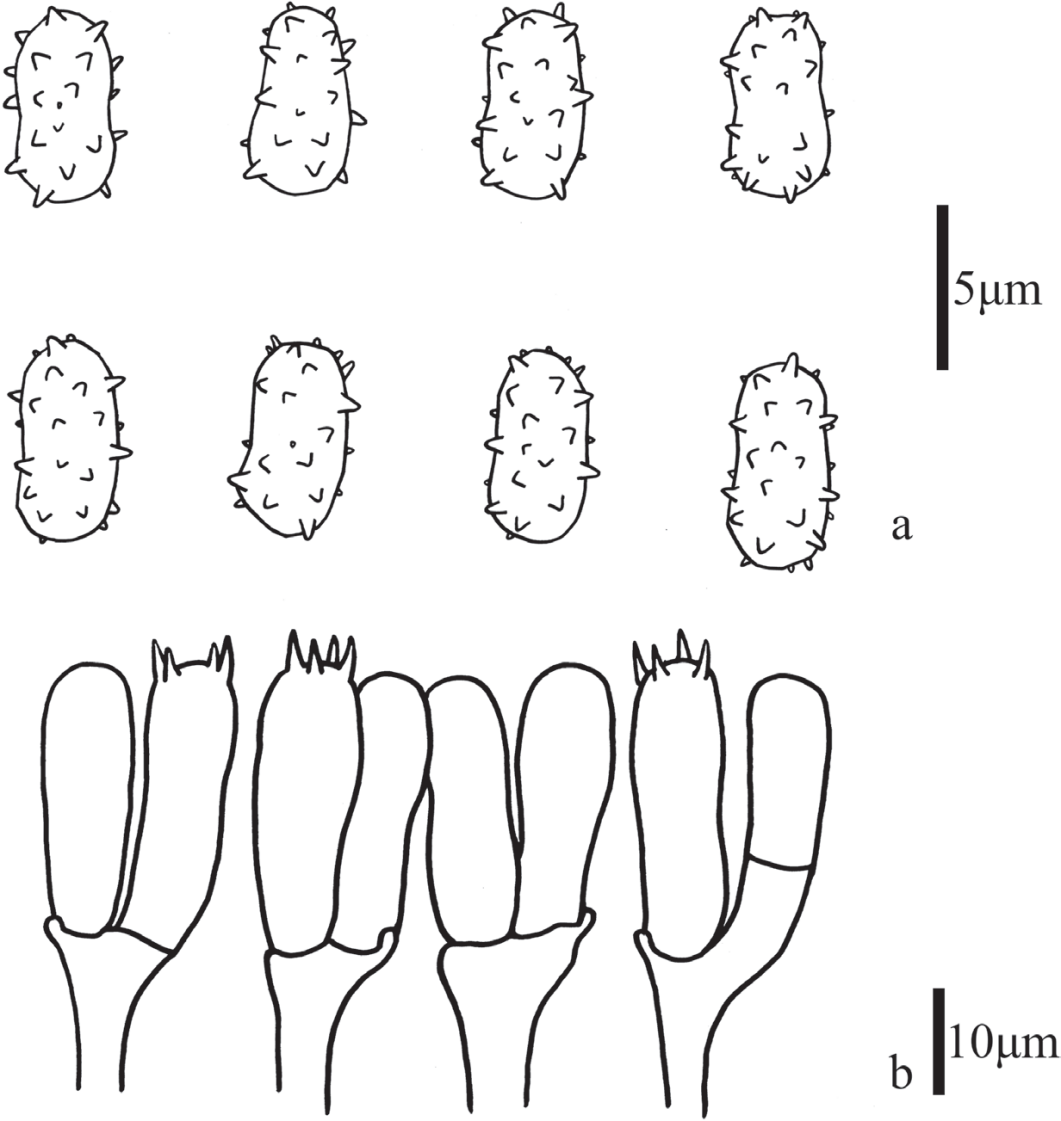
Microscopic features of *Trechisporalaxa* (MHHNU10714) **a** basidiospores **b** basidia.

##### Habit and distribution.

Solitary or scattered, grows in soil in broadleaf forest; basidiomata generally occur in summer. Known only from the type locality in China.

**Figure 7.**
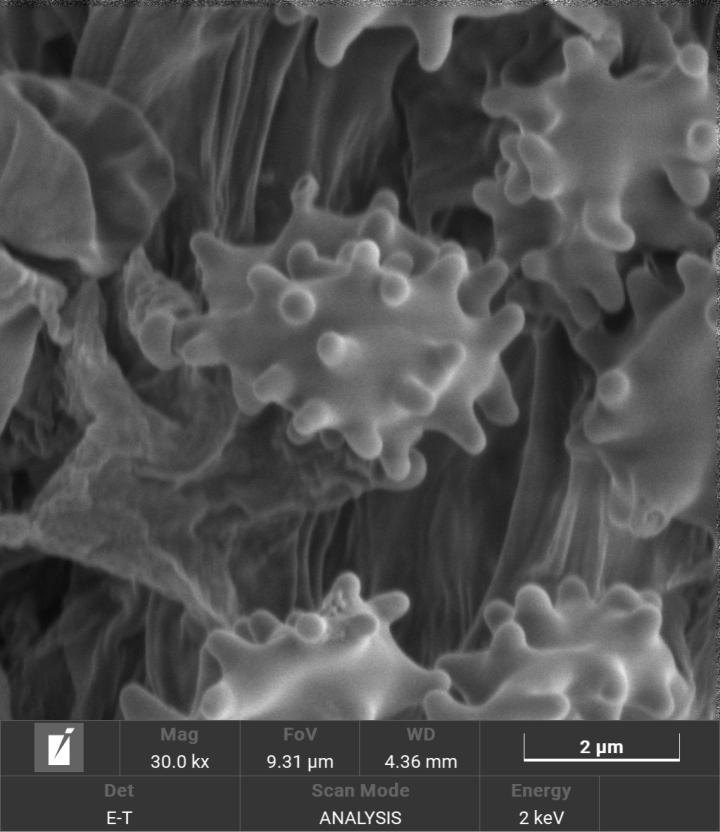
Scanning electron micrograph of basidiospores of *Trechisporalaxa* (MHHNU10714).

##### Notes.

The branches of *T.laxa* are scattered, not dense, and the apices are white or lilac gray with age. In the field, *T.laxa* and *Trechisporahavencampii* are similar because of their pale grayish brown coloration, but *T.havencampii* has dense branches, tips white and axils V-shaped, and 2-spored basidia with long sterigmata (5–9.5µm). *Trechisporalongiramosa* differs from *T.laxa* by having long terminal branches, densely branched and white to honey-yellow tips. *Trechisporasanpapaoensis* has smaller basidia (11–26 × 5.5–11.0 μm) and a grayish brown stipe. *Trechisporatermitophila* develops abundant basidiomata in active termite nests, but *T.laxa* generally grows in the soil of broadleaf forest. *Trechisporafoetida* has a reddish brown to deep brown, flattened stipe, and branching in one plane. In comparison, *T.laxa* is flesh colored, turning grayish purple in the terminal branches, the branches are not flattened, the branches are not white, and the stipe is non-flat. An additional pigmented species, *Trechisporarobusta* described from Brazil, is pale grayish with internodes irregular, branches flattened to subcylindrical, and inflated hyphae.

#### ﻿ Trechispora tongdaoensis

Taxon classificationFungiTrechisporalesHydnodontaceae

P. Zhang & P.T. Deng sp. nov.

DFDF06BA-CB3D-5783-B33D-EC0E19B42106

MycoBank No: 846849

Figs 8 , 9 , 10

##### Diagnosis.

Differs from *Trechisporatermitophila* by the white fruiting body and 4-spored basidia.

##### Type.

China, Hunan Province, Tongdao County, WanFo Mountain Nature Reserve, 26°32′54″N, 109°86′95″E, 523 m asl., 6 July 2022, leg. P. Zhang (holotype MHHNU11083).

##### Etymology.

*tongdaoensis* (Latin), referring to the currently known distribution of the species in Tongdao County, Hunan Province, China.

##### Basidiomata.

Clavarioid, gregarious to caespitose clusters, 60–90 mm tall, 30–45 mm broad, white (1A1), with pale yellow (1A3). Stipe single, 20–40 mm long, white (1A1), no change in color when dried. Branches dense, branching from the base, repeatedly dichotomous towards apices, divided 3–5 times, branches slender, 2–3 mm wide, internodes becoming gradually longer, terminal branches long and not flat, sometimes split at the tips, acute, axils V-shaped, terminal branches short, tips acute. Context pale yellow. Taste and odor not recorded.

**Figure 8.**
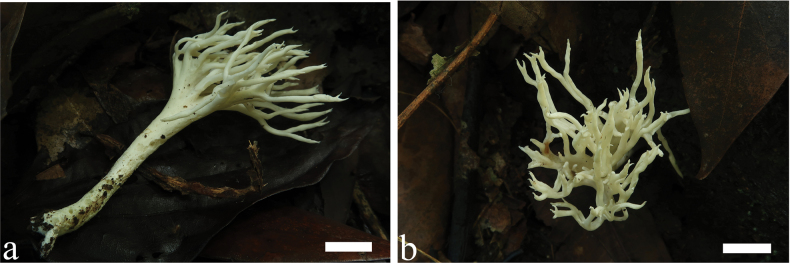
Basidiomata of *Trechisporatongdaoensis* (MHHNU11083). Scale bars: 1 cm.

##### Micromorphology.

Context hyphae compact, 3–5.5 μm wide, subparallel arranged, cylindric; generative hyphae with clamp connections but not at every septum, thin-walled, smooth, hyaline, no calcium oxalate crystals; ampullate septa present in the hyphae of the stipe, 7–8 µm wide. Basidia: approximately 18–28 × 6–8 µm with four sterigmata 3–5 µm long, hyaline, subclavate to cylindrical, with clamp connection in base. Cystidia absent. Basidiospores [40/3/2] 4–6(6.5) × 3–5.5 μm [Q = 1.25–1.57(1.83), Q_m_ = 1.50 ± 0.11] ellipsoid, slightly angular, tuberculate or coarsely echinulate, spines 0.5–1 μm long, blunt; hilar appendage ambiguous by spore ornamentation, sometimes contents uniguttulate, inamyloid.

**Figure 9.**
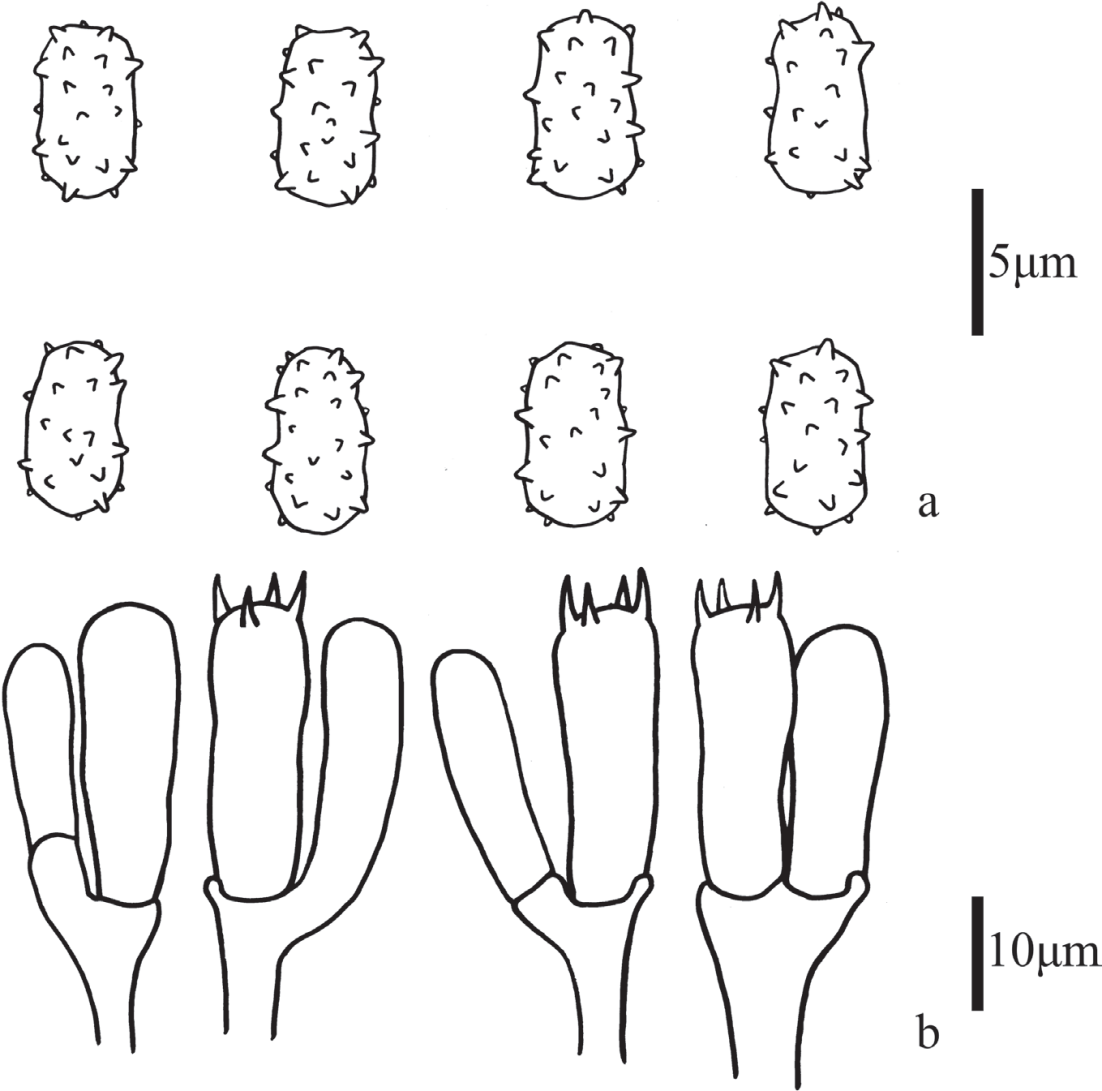
Microscopic features of *Trechisporatongdaoensis* (MHHNU11083) **a** Basidiospores **b** Basidia.

##### Habit and distribution.

Caespitose or gregarious on the soil of broadleaf forests; basidiomata generally occur in summer. Known only from the type locality in China.

**Figure 10.**
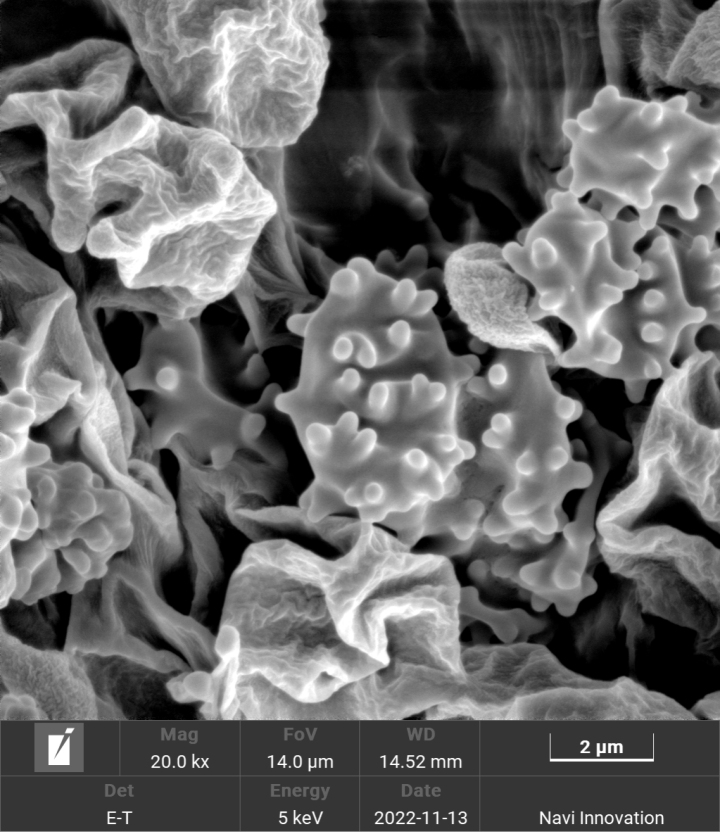
Scanning electron micrograph of basidiospores of *Trechisporatongdaoensis* (MHHNU11083).

##### Notes.

The fruiting body of *T.tongdaoensis* has a long stalk, 20–40 × 4–6 mm, the terminal branches are long, bifurcate, the tips are white, and the branches are not flattened in a plane. *Trechisporafoetida* differs from *T.tongdaoensis* by having reddish brown to deep brown basidiomata, and flattened branches and stipe. *Trechisporacaulocystidiata* has cystidia in the stipitipellis as caulocystidial hairs. This feature is obvious under a microscope, but we failed to observe this structure in *T.tongdaoensis*. In addition, the *T.caulocystidiata* stipe is relatively shorter (10–20 × 3–5 mm vs. 20–40 × 4–6 mm in *T.tongdaoensis*). *Trechisporacopiosa* differs from *T.tongdaoensis* in having acute or flattened branch tips and *T.tongdaoensis* has relatively smaller spores (4–6 (–6.5) × 3–5.5 μm vs. (5–) 5.5–6.5 (–7) × (3–) 3.5–4 (–4.5) μm in *T.copiosa*). *Trechisporadealbata* was classified in *Ramariopsis* (Donk) Corner based on the gelatinous context by Petersen (1984, 1988), whereas *T.tongdaoensis* lacks a gelatinous context and differs in having relatively smaller spores (4.0–4.5 × 2.5–3.5 µm vs. 4–6(6.5) × 3–5.5 μm in *T.tongdaoensis*). In the field, *T.gelatinosa* has a fleshy or gelatinous consistency, translucent when fresh, small basidia (12–26 × 5–6.5 μm), and small basidiospores (3–) 3.2–4.5 (–5) × (2–) 2.5–3.5 (–4) μm, which distinguishes the species from *T.tongdaoensis*. In the present phylogenetic analyses, *T.tongdaoensis* clustered with pigmented species.

## ﻿Discussion

*Trechispora* has until recently been considered to encompass a variety of morphological characteristics and broad diversity in hymenophore structure ranging from smooth, grandinioid to odontioid, hydnoid, or poroid, but always resupinate basidiomata. *Scytinopogon* is characterized by having clavarioid basidiomata and flattened branches, and the basidiomata are mostly white in color with a tough texture. Over time, additional pigmented species of *Scytinopogon* have been described and some species have rounded instead of flattened branches. A relationship between the flattened coralloid *Scytinopogon* and the resupinate *Trechispora* was first suggested by Jülich (1981), who placed the genera in separate families of the order Trechisporales, Hydnodontales. Subsequently, Meiras-Ottoni et al. (2021) analyzed a number of sequences from specimens of each genus, including the type species of each genus, and concluded that *Scytinopogon* is a synonym of *Trechispora*. In phylogenetic reconstructions, several transitions between fruiting body types are indicated to have occurred (Meiras-Ottoni et al. 2021). In addition, the color of *Trechispora* species has changed many times over the course of evolution, from white to pigmented, but the evolutionary trend for the basidiomata is not understood. The transition from resupinate basidiomata to clavarioid basidiomata seems to represent an evolutionary trend. The effect of this transformation is presumably an increase to expand the surface area of the hymenophore. The driving force behind morphological differences in fruiting bodies seems likely to be associated with selection for efficient spore dispersal (Hibbett and Binder 2002). In micromorphology, variation in basidiospores (smooth or ornamented) and basidia (2 or 4 sterigmata) represents different evolutionary directions, but the effects of these differences remain unknown. Most species of *Trechispora* are distributed in the tropics or subtropics, including coralloid species. Indeed, the two new species described in this study were collected in the subtropics of southern China. However, the current distribution of stipitate and clavarioid species does not include temperate regions. To comprehend the diverse factors contributing to the evolution in *Trechispora*, a number of specific aspects should be considered: substrate, nutritional mode, and environmental conditions. The ITS region is highly variable among *Trechispora* species and cannot be aligned reliably within the genus (Meiras-Ottoni et al. 2021), such that other genetic markers (e.g., LSU, SSU, ef1, rpb1, and rpb2) should be used to for phylogenetic analyses in *Trechispora*.

In China, previous studies have reported one new coralloid species of *Trechsipora* (*T.longiramosa*; Liu et al. 2022) and three species of *Scytinopogon* (*S.cryptomerioides*, *S.echinosporus* (Berk. and Broome) Corner and *S.pallescens*; Zhang and Yang 2003, Lin et al. 2022). The present phylogenetic analysis confirmed that *S.cryptomerioides* was firmly nested within *Trechispora*. Together with the previous studies mentioned here, this finding highlights that further exploration for coralloid species of *Trechispora* is needed. The present study expands our understanding of clavarioid species of *Trechispora* by providing descriptions and illustrations for two new species. The findings enrich our knowledge of the distribution of coralloid *Trechispora* species in China and the overall diversity of *Trechispora*.

### ﻿Key to coralloid *Trechispora* species in China

**Table:** 

1	Basidiomata pure white to pale yellow	**2**
–	Basidiomata grayish brown to pale purple	**4**
2	Basidiomata with no flattened branches	***T* . *tongdaoensis***
–	Basidiomata with flattened branches	**3**
3	Basidiomata only grow in soil	***T.pallescens***
–	Basidiomata grow in the humus layer on soil	***T* . *khokpasiensis***
4	Basidiomata with dense branches and long terminal branches	***T.longiramosa***
–	Basidiomata with loose branches	***T* . *laxa***

## Supplementary Material

XML Treatment for Trechispora khokpasiensis

XML Treatment for Trechispora laxa

XML Treatment for Trechispora tongdaoensis

## ﻿Additional information

### Author ORCIDs

Peng-Tao Deng https://orcid.org/0000-0002-8755-7965

Jun Yan https://orcid.org/0000-0002-2832-8046

Zheng-Mi He https://orcid.org/0000-0001-8754-3427

Ping Zhang https://orcid.org/0000-0002-8751-704X

### Data availability

The sequence data generated in this study are deposited in NCBI GenBank.

## Supplementary materials

Supplementary material 1 Multiple sequence alignment Data type fasta file https://binary.pensoft.net/file/902757This dataset is made available under the Open Database License (http://opendatacommons.org/licenses/odbl/1.0/). The Open Database License (ODbL) is a license agreement intended to allow users to freely share, modify, and use this Dataset while maintaining this same freedom for others, provided that the original source and author(s) are credited.Peng-Tao Deng, Jun Yan, Xiang-Fen Liu, Zheng-Mi He, Yuan Lin, Ming-Xin Lu, Ping Zhang
